# Hip Geometry and Proximal Femoral Fractures among Elderly Filipino Women: A Single Centre Cross-Sectional Study

**DOI:** 10.5704/MOJ.2207.009

**Published:** 2022-07

**Authors:** CI Barrido, JAM Bengzon

**Affiliations:** Department of Orthopaedics, Philippine Orthopedic Center, Quezon City, Philippines

**Keywords:** hip geometry, proximal femur, femoral neck, intertrochanteric, risk factor

## Abstract

**Introduction:**

Few controlled studies explore proximal femoral geometry and association with femoral neck (FN) or intertrochanteric (IT) fractures, especially among the elderly Filipino population. Previous reports, however, still reveal multiple inconsistencies. The objective of the study is to establish a possible association between radiographic hip geometry and proximal femoral fractures based on measurements taken from elderly Filipino women.

**Materials and methods:**

This is a cross-sectional study of 182 Filipino women ≥ 60 years old at a single institution last 2019-2020. Patients were divided into groups with femoral neck fractures (n=84), intertrochanteric fractures (n=64), and those without diagnosed hip fractures (n=34). Standard pelvic radiographs with control of hip internal rotation was done and the following radiographic parameters were compared: hip axis length (HAL), femoral neck length (FNL), neck shaft angle (NSA), horizontal offset (HO), femoral head diameter (FHD), and femoral neck diameter (FND).

**Results:**

Data suggests that an increased FND increased the risk for acquiring both femoral neck (OR = 1.31, 95% CI 1.06 - 1.62; p=.011) and intertrochanteric fractures (OR: 1.22, 95% CI 1.07-2.16; p=0.018). For intertrochanteric fractures alone, a wider NSA (OR 1.27, 95% CI 1.02 - 1.58, p=0.033) and larger HO (OR 1.29, 95% CI 1.02 - 1.64, p=0.036) also increased the risk for this fracture type while a longer HAL was protective (OR 0.85, 95% CI 0.73 - 0.98, p=0.30). Other radiographic parameters and ratios revealed no association.

**Conclusion:**

Results show that there are certain hip geometric parameters that play a role in the risk and incidence of developing femoral neck or intertrochanteric fractures. These measurements may aid in identification of patients at risk. This study may act as a guide for future implant design and increase accuracy of hip reconstruction among elderly Filipino women.

## Introduction

Incidence of hip fractures continuously increases with age and is the leading cause of morbidity and mortality. In an observational cohort study by Roche *et al*^1^, the researchers expect hip fractures, being a common complication of osteoporosis, to affect 6.3 million individuals worldwide by 2050, including 3.25 million in Asia. With a high mortality rate of 20% - 33% one year after hip fracture occurrence^2,3^, high level studies evaluating other relevant risk factors for hip fractures are needed.

There are currently more than six million Filipinos greater than 60 years old (6% - 7% of total population) and this is expected to reach 26 million (17.9% of expected population) by the year 2050 according to the International Database by the U.S. Census Bureau. A local study done at a single Philippine institution last 2008 estimated the prevalence of hip fractures in individuals 70 years and above to be 160 per 10,0004. From 2012-2017, the same institution admitted an average of 367 patients per year with femoral neck fractures and 295 patients per year with intertrochanteric fractures. Hip fractures among Filipinos are expected to reach 65,000 by year 2020 and 175,000 by year 2050. With osteoporosis being a widely accepted factor contributing to incidence of these hip fractures, it is forecasted that more than 10 million people will be high risk by 20505.

Mechanical strength of a bone is related to physical qualities of its materials (volume of material and their type of special dispersion), geometry, and conditions including direction and amount of the force applied^6-8^. Non-geometric risk factors for hip fractures have been extensively investigated.

Among these factors include advanced age, female sex, osteoporosis, genetic factors (e.g. colia1 sp1 polymorphism), smoking, alcohol abuse, previous fractures, and low oestrogen levels^9-12^. It is widely accepted that these non-geometric factors, especially lower bone mineral density and increased tendency to fall, are proven to be related to hip fracture incidence. These alone, however, do not sufficiently explain all that may be attributed to hip fractures which therefore encourages further research focusing on the geometric aspect^13^. Hip geometry deals with the investigation of the shape and anatomic dimensions of the proximal femur currently a topic of interest.

There are few local studies attempting to get dimensions of the Filipino bone architecture. Filipino femoral head size and correlation to height and weight was explored by Suntay last 198814. Spinal canal measurements among Filipinos were measured by Mariano last 198215. Guloy and Apolinario, determined the average dimensions of adult Filipino femora (mean age 49 years old) among 15 cadaveric specimens in two studies last 1990 and 1991^16-17^. Paucity of accurate data on the relation of proximal femoral geometry to increased risk of hip fractures, especially among the elderly Filipino population, is evident. Standardised pelvis radiographs, including supervised patient positioning and adjustment for magnification, may produce more plausible findings which differs this to previous retrospective studies. The lack of baseline data and consensus warrants further similar higher-level studies.

The objective of this study was to establish if there is an association between radiographic hip geometry and risk for proximal femoral fractures based on average measurements taken from elderly Filipino women. Specific objectives were the following: (1) to determine average radiographic hip dimensions (HAL, FNL, NSA, HO, FHD, FND, FHNO) of Filipino women ≥60 years old in a single Philippine institution, (2) to compare radiographic hip dimensions among Filipino women ≥60 years old with and without hip fractures (femoral neck and intertrochanteric) and (3) to identify radiographic hip dimensions increasing risk for specific proximal femoral fracture types.

## Materials and Methods

This is an epidemiological, observational, analytical, and cross-sectional study. Possible confounders or effect modifiers include age, BMI, bone mineral density, genetic factors, smoking, alcohol abuse, oestrogen levels, and metabolic bone disease. Considering the local hospital census and regional data, a minimum of 102 subjects, or 34 patients per group (3 groups), was required for this study based on a level of significance of 5% and a power of 90%, based on the study by Partanen *et al* last 200118.

Filipino female ≥ 60 years old who sought consult at the outpatient department (OPD) of a single institution last 2019-2020 were included. Patients with the following were excluded: fractures from severe trauma (e.g. car accidents); bilateral hip fractures; subtrochanteric fractures; diagnosed malignancies; rheumatologic disease (e.g. renal, hepatic, pulmonary); diagnosed thyroid disease; paralysis; history of stroke; previous intake of drugs known to affect bone (e.g. oestrogen, steroids, calcitonin, bisphosphonates, anti-epileptic drugs); poor quality radiographic imaging (including patients with contractures or limited range of motion of extremities); radiographic evidence of hip osteoarthritis, subluxation, or dislocation.

Quota sampling was done and the achieved recruited patients (n=182) exceeded the minimum required subjects to meet the desired statistical constraints. Subjects were categorised into the following groups: without hip fractures (n=34), previously diagnosed with femoral neck fractures (n=84), and previously diagnosed with intertrochanteric fractures (n=64). All initial consults at the institution were due to a history of fall from standing height. Informed consent was obtained, and baseline demographics taken. Confirmation of hip fracture and identification of type was done from previous documented radiographs. The need for the pelvis radiograph was pre-determined by the patient’s attending physician in-charge during time of consult.

**Fig. 1:**
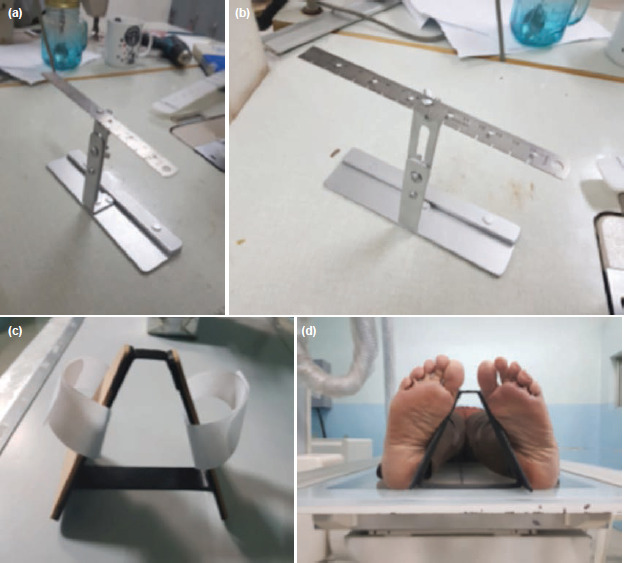
(a, b) Radiographic ruler with adjustable height, (c, d) 20° internal rotation foot angle guide with base at 20cm apart.

Standard pelvis AP radiographs were taken at the institution’s radiology department with machines pre-calibrated and positioning supervised by trained radiology technologists who attended a briefing regarding the study protocol. Positioning of the patient, radiograph beam, receptor, and collimator were standardised and patterned after that described by Long *et al* (2016)^19^. A custom prefabricated foot angle guide was used providing 20° internal rotation of both lower extremities and keeping a 20cm distance between both heels. The medial aspects of the feet were rested on the guide and held in place with a velco strap during the procedure. To account for magnification, patterned after the coin method by Conn *et al*^20^, a radiographic ruler with pre-determined measurements was placed at the level and height of the greater trochanter (Fig. 1). Radiographs were evaluated in terms of quality by the researcher prior to inclusion of a subject in the study.

**Fig. 2:**
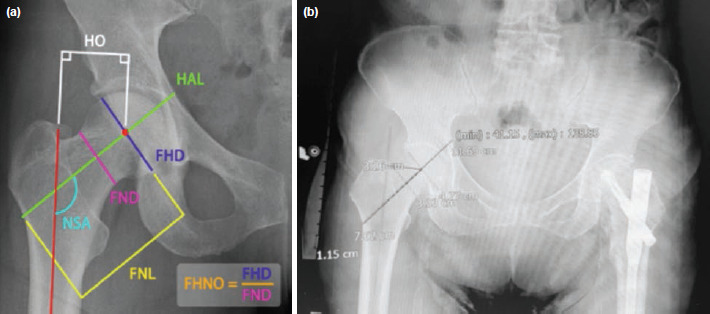
(a) Schematic diagram of radiographic measurements. (b) Actual sample radiograph and measurement method.

Radiographic geometry of the of the non-fractured hip was assessed using the local Picture Archiving and Communication System (PACS) viewer by a single researcher, an orthopaedic surgeon, using fixed anatomic points and landmarks determined with digital imaging. The following parameters and ratios were recorded (Fig. 2): Hip axis length (HAL) - inner pelvic brim to the lateral side of the femur, Femoral neck length (FNL) - length between lateral cortex in proximal femur and centre of femoral head along femoral neck axis, Neck shaft angle (NSA) - angle formed by the mid-cervical axis and the mid-shaft axis, Horizontal offset (HO) - distance of head centre from mid-shaft axis, Femoral head diameter (FHD) - femoral head diameter drawn from centre of femoral head, and Femoral neck diameter (FND) - longest femoral neck diameter.

Descriptive statistics was used to summarise the general and clinical characteristics of the participants. Frequency and proportion were used for nominal variables, median and range for ordinal variables, and mean and standard deviation for interval/ratio variables. One-way ANCOVA, Kruskal-Wallis test and Fisher’s Exact test was used to determine the difference of mean, median and frequency, respectively. Odds ratios and the corresponding 95% confidence intervals from binary logistic regression was computed to determine the association between hip dimensions and risk for femoral neck and intertrochanteric fractures. Protocol approved by local institution ethical board.

## Results

A total of 182 patients were included in the study (Table I), with a median age of 74.5 years (ranges from 60 to 96 years). Laterality was comparable across the groups (p=.507). As to age, those in the intertrochanteric group were the eldest among the three (median 79 vs 69.5 and 72 years, p=.0001). Mean magnification was at 14 percent (±0.03 standard deviation) and was similar across groups (p=.291).

**Table I:** Demographic profile of patients (n=182)

	Total (n=182)	No fracture (n=34)	FNF (n=84)	ITF (n=64)	P
		Frequency (%); Mean±SD			
Age, years	74.5 (60-96)	69.5 (63-86)	72 (60-92)	79 (60-96)	.0001*
Laterality					.507†
Left	74 (50.00)	-	44 (52.38)	30 (46.88)	
Right	74 (50.00)	-	40 (47.62)	34 (53.13)	
Magnification	1.14±0.03	1.14±0.03	1.15±0.03	1.14±0.03	.291*

Abbreviations; FNF: femoral neck fracture, ITF: intertrochanteric fracture

Statistical tests used; *, Kruskal-Wallis test; †, Chi-square test

Using the one-way ANCOVA test with age as the covariate, the three groups were significantly different in terms of femoral neck diameter (p=¬.033), with the widest mean diameter noted in the femoral neck fracture group (Table II). In comparison to the control group, those under the femoral neck group also had a significantly larger FND with a mean difference of 0.10 (ranges 0.02 to 0.18, p=.046). (Table III). Our results suggest an association between FND and femoral neck fractures (Table IV). A 1mm increase in FND is associated with a 31% increase in odds of having a femoral neck fracture (OR = 1.31, 95% CI 1.06 to 1.62; p=.011). This model explains 6.61% in the variation of femoral neck fractures (p=0.0092).

**Table II:** Radiologic hip geometry of patients (n=182)

	**Total (n=182)**	**No fracture (n=34)**	**FNF (n=84)**	**ITF (n=64)**	**P**
		**Frequency (%); Mean±SD**			
HAL, cm	10.38±0.56	10.34±0.65	10.43±0.55	10.33±0.53	.580
FNL, cm	6.78±0.42	6.76±0.47	6.76±0.41	6.82±0.39	.758
NSA, degrees	134.54±4.19	134.16±4.08	134.58±4.39	134.69±4.05	.868
HO, cm	3.27±0.44	3.25±0.45	3.25±0.49	3.31±0.36	.939
FHD, cm	4.31±0.23	4.28±0.21	4.33±0.22	4.30±0.25	.484
FND, cm	2.93±0.20	2.85±0.23	2.95±0.19	2.94±0.19	.033
FHD/FND	1.48±0.10	1.51±0.10	1.47±0.08	1.47±0.11	.107
HAL/FND	3.56±0.24	3.65±0.25	3.55±0.25	3.52±0.21	.080
FNL/FND	2.33±0.19	2.39±0.23	2.31±0.19	2.32±0.15	.081
HAL/HO	3.23±0.43	3.23±0.41	3.28±0.50	3.15±0.34	.471

*Abbreviations*; FNF: femoral neck fracture, ITF: intertrochanteric fracture, HAL: hip axis length, FNL: femoral neck length, NSA: neck shaft angle, HO: horizontal offset, FHD: femoral head diameter, FND: femoral neck diameter

Statistical test used; One-way ANCOVA (with age as covariate)

**Table III:** Multiple comparisons test for femoral neck diameter

	Mean difference (95% CI)	P
FNF vs Control	0.10 (0.02 to 0.18)	.046
ITF vs Control	0.10 (0.01 to 0.19)	.066
ITF vs FNF	0 (-0.07 to 0.07)	.998

Abbreviations; FNF: femoral neck fracture, ITF: intertrochanteric fracture

Statistical test used; Scheffe’s test

**Table IV:** Predictors of femoral neck and intertrochanteric fractures

	Mean difference (95% CI)	P
Femoral Neck Fractures:		
FND	1.31 (1.06 to 1.62)	.011
Intertrochanteric Fractures:		
HAL	0.85 (0.73 to 0.98)	.030
FND	1.22 (1.07 to 2.16)	.018
NSA	1.27 (1.02 to 1.58)	.033
HO	1.29 (1.02 to 1.64)	.036

Abbreviations; FND: femoral neck diameter, HAL: hip axis length, NSA: neck shaft angle, HO: horizontal offset Adjusted model R2=6.61%; p=.0092 (femoral neck fractures)

Adjusted model R2=22.90%; p <.0001 (intertrochanteric fractures)

HAL, FND, NSA, and HO were significantly associated with intertrochanteric fractures (Table IV). A 1mm increase in HAL is associated with a 15% decrease in odds of having an intertrochanteric fracture. Meanwhile, a 1mm increase in FND, NSA, and HO are associated with increased odds of having intertrochanteric fractures. This model explains 22.90% in the variation of intertrochanteric fractures (p <0.0001).

## Discussion

The main difference of this study to previous publications was the utilisation of standardised pelvic radiographs done with a controlled hip position using a prefabricated foot angle guide and a radiographic ruler to adjust for magnification. To the author’s knowledge, focus and careful critique of the quality of each radiograph prior to measurement provided more credibility to the study.

The FND was noted to be largest in patients with a femoral neck fracture compared to other groups. A larger FND increased the risk of acquiring either a femoral neck or intertrochanteric fracture. An increase in NSA and HO was associated with a higher risk of intertrochanteric fractures, while a longer HAL was protective for the same group.

One-millimeter increase in FND was found to be associated with a 31% increase in odds of a femoral neck fracture as well as a 22.9% increase of odds for an intertrochanteric fracture. This coincides with the results from a meta-analysis by Fajar *et al* last 2017^21^ comprising of 11 studies from published international literature up until May 25th, 2017, as well as the study by Michelotti and Clark, last 1999^22^ comparing radiographic femoral neck measurements of 119 American patients. One possible explanation for the results as stated by Michelotti and Clark is that there is a correlation of osteoporosis and overall dilatation of the femur, which may be simply associated to aging^22^. It is found that the upper femoral neck cortex thins out and expands with age, thus weakening the femoral neck, while the thickness of the lower femoral neck cortex rarely changes^23^.

An increase in NSA also showed higher odds of acquiring an intertrochanteric fracture in our study. In Italy, Gnudi *et al*^24^ published a 5 year follow-up of 729 post-menopausal Italian women and noted that hip fracture incidence was higher among females with a wide NSA (8.52%) when compared to those with a narrow NSA (3.51%). Proximal hip geometry was reviewed by Im and Lim^25^ via radiographs among 151 Koreans last 2005-2009 showing similar results of an increased risk for intertrochanteric fractures 2.32-fold with each 1 standard deviation (SD) increase in NSA. A larger NSA would create a greater force absorbed by the proximal femur when falling on the lateral side because of a longer moment arm as reinforced by Wang *et al*^26^. An opposing study last 2018 on 198 elderly Chinese showed no correlation of the NSA to incidence of intertrochanteric fractures, however, they found that a greater NSA increased the risk of acquiring femoral neck fractures^27^. The previously mentioned meta-analysis by Fajar *et al*^21^ showed similar results declaring that patients with femoral neck fractures had a significantly larger NSA compared to other hip fracture types. They however admitted multiple limitations to their study including false positive findings due to small sample sizes and excluded investigation of other factors such as age, gender, ethnicity, menstrual status, osteoporosis, and drug use. Few other studies concluded NSA had no correlation to incidence of hip fractures^28-30^.

An increased horizontal offset (HO) increased the risk of intertrochanteric fractures in our study. Most previous publications excluded measurement of this parameter, maybe due to the wide variations that came with a small change in hip position. An internally rotated hip will portray a smaller HO compared to one that is externally rotated. Inconsistent with our results, Im and Lim^25^ found that patients with femoral neck or intertrochanteric fractures had a lower HO compared to the control, but results were insignificant.

A larger hip axis length (HAL) was protective in terms of risk for intertrochanteric fractures. The data of our study showed a significantly older population among the intertrochanteric group (median of 79 years) compared to the control and femoral neck group. Radiographs of this population may present with a narrower joint space due to age, therefore decreasing overall HAL and possibly increasing chance for falls. An ex-vivo biomechanical test by Cheng *et al*^31^ supported our findings by demonstrating that femoral neck strength was greater in hips with longer HAL, implying that fractures may be less likely in such subjects. Gnudi *et al*^32^ indicated that women with trochanteric fractures had relatively small HAL and NSA. These results are opposite to the findings by Im and Lim^25^ declaring that every 1 SD increase of HAL increased the risk for intertrochanteric fractures 1.64-fold.

The varied conclusions among previous studies may be due to different protocols of imaging and measurement, limitation in sample size, and conduction among different ethnic populations. The results of this study may be the first that utilised data from a more standardised method of taking pelvic radiographs, control of internal rotation of the hip using a guide, and adjustment of magnification using a radiographic ruler. To the researchers’ knowledge, this is the first study regarding hip geometry and fracture risk in the Philippine population and is among one of the few local studies concerning average dimensions of the adult Filipino femur. In comparison to data from other countries, it is evident that there is a wide variation per race. These average measurements may be considered during internal fixation as well as hip reconstruction.

Limitation of our study is that we utilised two-dimensional radiographs for feasibility. It would be ideal to apply 3D computed tomography (CT) for increased accuracy, however, this would not be cost-effective and may deliver a large unnecessary radiation dose to patients. All patients in this study presented with a low-energy injury caused by a fall from standing height. Other factors such as height, weight, BMI, and bone mineral density were not included but targeted sample size of the study was exceeded to provide a general depiction of the study population, specifically the elderly Filipino females of lower socio-economic class within the region. A prospective randomised cohort with a larger sample size, multiple data collectors to perform repeated measurements increasing intra and inter-observer reliability, and analysis of other contributing factors may be recommended.

## Conclusion

In conclusion, disparities in radiographic hip parameters among elderly Filipino women as well as risk for different hip fracture types are apparent. The major risk factor identified for acquiring femoral neck fractures was a larger FND. For intertrochanteric fractures, risk factors included a large FND, wide NSA, and a large HO, while a longer HAL was protective. These findings suggest hip geometry may play a significant role in the incidence of certain hip fracture types as well as guide future implant design tailored for the elderly Filipino population.
